# Microbial infections in HIV/AIDS women with abnormal vaginal discharge in Lagos, Nigeria

**DOI:** 10.1186/1471-2334-14-S2-P40

**Published:** 2014-05-23

**Authors:** NM Otuonye, SI Smith, NN Odunukwe, CT Oparaugo, AA Adesesan, RN Okoye, CV Gab-Okafor, RC Chigbo

**Affiliations:** 1 Nigerian Institute of Medical Research, Lagos, Nigeria

## Introduction

Vaginal infections which are caused by bacterial vaginosis (BV), bacteria pathogens (BP) trichomoniasis (TV), and yeast infections are common among HIV-infected women. This may be a marker for increased transmissibility to sexual partners, infants at delivery, significant morbidity, underscoring their importance from a public health perspective.

## Methods

Three eighty seven (387) patients who presented to the HIV clinic with symptoms of lower abdominal pain, itching and abnormal vaginal discharge were selected after obtaining written informed consent. Patients on oral or vaginal medications for vaginitis were excluded. High vaginal/cervical swabs were collected, cultured and processed using standard microbiological methods.Anti-microbial sensitivity patterns of the isolates were determined. The characteristics of the discharge, vaginal pH >4.5, presence of ‘clue cell’ and Amine test with 10% KOH were used for Bacterial vaginosis (BV) investigations. The age range of study population was between 20 – 45 years with mean of 24+. All patients complained of abnormal vaginal discharge.One hundred and twenty (38.46%) had lower abdominal pain, itching / irritation 200 (64.10%) and 30 (9.61%) had sore and blisters on the genitals. Vaginal pH > 5.0 was recorded in 215 (68.91%) of the patients.

## Results

A total of 80.6% of HIV/AIDS women were infected withmicrobial infection.Microbial agents isolated were as follows: Candida species 163 (52.2%), BV 77 (24.6%), bacterial pathogens 66 (21.2%) and Trichomonasvaginalis 6 (2.0%).Thirty bacterial isolates co-infected with Candidaspecies while 3 T. vaginalis co-infected with Candida species, 15 BV co-infected with other bacterial pathogens. About 4 patients had triple infection of BV, yeast and bacterial pathogens.

## Conclusion

Most of the bacterial isolates were sensitive to Ciprofloxacin, Ofloxacin, levofloxacin and gentamicin antibiotics.Microbial infections in HIV/AIDS women was statistical significant (p>5.0). Treating an HIV positive woman presenting with abnormal vaginal discharge would reduce transmission of HIV virus to her sexual partners and perinatal HIV transmission.

